# Initial Denosumab Versus Sequential Bisphosphonate-to-Denosumab for Prevention of Skeletal-Related Events in Breast Cancer with Bone Metastases: A Retrospective, Single-Center Study

**DOI:** 10.3390/cancers18081222

**Published:** 2026-04-12

**Authors:** Yannan Zhao, Bo Yu, Wanjing Feng, Yizhao Xie, Yuanyuan Shi, Jun Cao

**Affiliations:** Department of Medical Oncology, Fudan University Shanghai Cancer Center, Shanghai 200032, China

**Keywords:** breast cancer, bone metastases, bone-targeted agent, denosumab, bisphosphonates, skeletal-related events (SREs), sequential therapy, retrospective study, real-world evidence

## Abstract

**Background:** Skeletal-related events (SREs), including pathological fractures, spinal cord compression, radiotherapy to bone, and bone surgery, substantially worsen quality of life in breast cancer with bone metastases. Denosumab, a monoclonal antibody targeting RANKL, mechanistically differs from bisphosphonates and is not renally cleared, offering potential clinical advantages. In practice, an increasing number of patients transition from bisphosphonates to denosumab. However, the comparative effectiveness of sequential therapy versus initial denosumab remains unclear. **Methods:** We retrospectively analyzed 165 patients with breast cancer and radiologically confirmed bone metastases treated between 1 January 2019 and 30 April 2024 at a tertiary center in China. Patients were categorized into an initial denosumab group (n = 67) or a sequential bisphosphonate-to-denosumab group (n = 98). The primary endpoint was time to first on-treatment SRE; the 12-month first on-treatment SRE rate was also reported as a descriptive summary measure. Secondary endpoints included cumulative SRE incidence and safety. Kaplan–Meier and log-rank tests compared SRE-free survival; Cox regression explored prognostic factors. **Results:** The median age at bone-metastasis diagnosis was 54.7 years. Median time from diagnosis to bone-targeted agents (BTAs) initiation was 0.9 months in both groups; median follow-up was longer in the sequential group (22.5 vs. 11.3 months). At diagnosis, 46 of 165 patients (27.9%) presented with synchronous SREs, more frequent in the initial denosumab group (37.3% vs. 21.4%; *p* = 0.040). During follow-up, 31 patients (18.8%) developed SREs: 25 of 98 (25.5%) in the sequential group versus 6 of 67 (9.0%) in the initial denosumab group (*p* = 0.008). After BTA initiation, on-treatment SREs occurred in 28 of 165 patients (17.0%): 25 of 98 (25.5%) in the sequential group versus 3 of 67 (4.7%) in the initial denosumab group (*p* < 0.001). The 12-month first on-treatment SRE rate was 15.7% (95% CI 8.1–22.7) for sequential therapy and 5.9% (0–12.3) for initial denosumab. In Cox analysis, second-line systemic therapy increased SRE risk (HR = 2.651, *p* = 0.021). Safety outcomes were generally manageable and consistent with known class effects, with no clear exposure-adjusted safety advantage of one strategy over another. **Conclusions:** Initial denosumab was associated with fewer and delayed SREs compared with sequential bisphosphonate-to-denosumab therapy, supporting early denosumab initiation as a potentially preferable BTA strategy. Prospective studies are warranted to confirm these findings.

## Simple Summary

Bone metastases are frequent in advanced breast cancer and can cause skeletal-related events (SREs), such as pathological fractures or the need for radiotherapy or surgery on bone. Bone-targeting agents reduce these complications, but in routine practice some patients start with bisphosphonates and later switch to denosumab; whether this sequential strategy performs similarly to upfront denosumab remains uncertain. We retrospectively analyzed 165 patients with breast cancer and radiologically confirmed bone metastases treated at a single tertiary center (2019–2024), comparing initial denosumab (n = 67) with bisphosphonate-to-denosumab sequential therapy (n = 98). On-treatment SREs occurred less often in the initial denosumab group (4.7% vs. 25.5%), and the estimated 12-month first on-treatment SRE rate was numerically lower (5.9% vs. 15.7%). Overall, our real-world data supports considering denosumab at diagnosis of bone metastasis to reduce early skeletal complications, pending prospective confirmation.

## 1. Introduction

Breast cancer is one of the most common malignancies worldwide. According to the GLOBOCAN 2022 estimates, female breast cancer was the second most frequently diagnosed cancer after lung cancer, with approximately 2.31 million new cases, accounting for 11.6% of all cancers in 2022. It is also a major cause of cancer-related death, ranking fourth globally with an estimated 665,684 deaths, corresponding to 6.9% of all cancer deaths in the same year [1]. The skeleton is the predominant site of distant metastasis in breast cancer; nearly 70% of patients who die from the disease have evidence of bone metastases at autopsy [2]. Bone is therefore the most frequent site of distant spread and constitutes the initial metastatic site in approximately 25–40% of patients with advanced disease [3].

The natural history of breast cancer bone metastases is dominated by osteoclast-mediated bone destruction and the development of skeletal-related events (SREs)—pathological fractures, spinal cord compression, and the need for radiotherapy or surgery to bone, often accompanied by tumor-related hypercalcemia [4,5]. These events are the principal drivers of pain, impaired mobility, and neurological compromise, and they substantially worsen quality of life, increase hospitalizations, and impose a considerable economic burden on patients and health systems [6]. Preventing or delaying SREs has therefore become a major therapeutic objective in the management of metastatic breast cancer [4].

Bone-targeting agents (BTAs) have been the cornerstone of supportive care in this setting for more than two decades. Intravenous bisphosphonates, such as zoledronic acid and ibandronate, inhibit osteoclast-mediated bone resorption and reduce the risk of SREs, but their use is limited by the need for renal monitoring, infusion-related toxicity, and incomplete protection against events [7,8]. Denosumab, a fully human monoclonal antibody directed against receptor activator of nuclear factor-κB ligand (RANKL), produces potent and reversible suppression of osteoclast differentiation and survival and is not renally cleared [5,8,9]. Recent clinical and economic evaluations indicate that denosumab provides modest but clinically relevant gains in preventing and delaying SREs, with less renal toxicity but more hypocalcemia compared with zoledronic acid [6,8]. On this basis, contemporary international guidelines recommend long-term treatment with a bone-modifying agent, either denosumab or a potent bisphosphonate, for patients with advanced breast cancer and bone metastases, unless contraindicated [4,9].

Despite this evidence, real-world practice often deviates from guideline recommendations. In many regions, bisphosphonates remain the initial bone-targeted agents because of availability, cost considerations, or prescribing habits, and patients are subsequently switched to denosumab after months of bisphosphonate exposure [6,10,11,12,13]. This sequential bisphosphonate-to-denosumab strategy is increasingly common, but its effectiveness and safety compared with initial denosumab initiation remain uncertain, because most available data derive from observational series or mixed solid-tumor cohorts rather than dedicated comparative studies [14,15,16].

Randomized trials have provided head-to-head comparisons of denosumab and zoledronic acid, showing superior prevention of skeletal-related events with denosumab, but these studies evaluated de novo initiation and therefore do not directly address the clinical outcomes of sequential therapy [16]. Moreover, much of the evidence base comes from controlled trial populations, whereas contemporary practice involves more heterogeneous patients with diverse tumor subtypes, prior treatments and comorbidities [4,10,11,13].

Clarifying whether initial denosumab provides superior protection against SREs compared with sequential bisphosphonate-to-denosumab therapy is clinically important, as it may guide optimal BTA choice at the time of bone-metastasis diagnosis and help avoid preventable morbidity. To address this knowledge gap, we conducted a retrospective cohort study of 165 patients with breast cancer and radiologically confirmed bone metastases treated at a single tertiary cancer center in China. We compared the incidence and timing of SREs, as well as the profile of treatment-related adverse events, between patients who received denosumab as initial BTA therapy and those who started on bisphosphonates and subsequently switched to denosumab.

## 2. Methods

### 2.1. Patients

**Figure 1:**
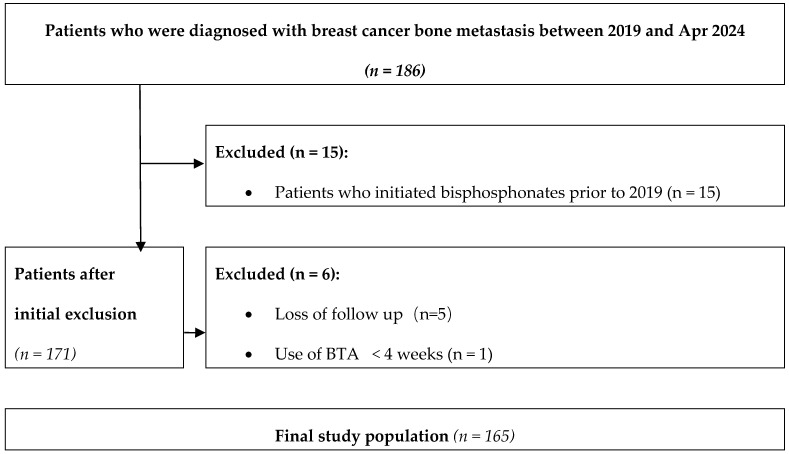
Flowchart of patient screening and cohort selection. Patients diagnosed with breast cancer bone metastases between January 2019 and April 2024 were assessed for eligibility. Patients who initiated bisphosphonate treatment before 2019, were lost to follow-up, or received bone-targeting agents for less than 4 weeks were excluded before defining the final analytical cohort.

We conducted a retrospective, single-center cohort study at Fudan University Shanghai Cancer Center (Shanghai, China). Eligible participants were women aged 18 years or older with histologically confirmed breast cancer and radiologically verified bone metastases who received bone-targeting agents between 1 January 2019 and 30 April 2024. We initially identified 186 patients. To ensure a uniform treatment era in which denosumab was available, 15 patients treated before 2019 were excluded because denosumab had not yet been approved in China at that time. Furthermore, 6 patients who received only a single day of denosumab treatment were excluded. After these exclusions, 165 patients were included in the final analysis, comprising 98 treated with sequential bisphosphonate-to-denosumab therapy and 67 treated with denosumab alone (Figure 1).

Baseline demographic and clinical data were extracted from electronic medical records, including age at breast cancer and bone-metastasis diagnosis, menopausal status, tumor subtype (hormone-receptor and HER2 status), disease stage, presence of visceral metastases, number and sites of bone lesions, systemic therapy history, and prior local treatments. Patients were categorized according to BTA strategy into two groups: those who received denosumab as first-line bone-targeted therapy (initial denosumab group) and those who received prior bisphosphonate therapy before subsequent switching to denosumab (sequential bisphosphonate-to-denosumab group). In the sequential group, documented prior bisphosphonate exposure before denosumab was required for classification.

### 2.2. Treatment and Follow-Up

Denosumab was administered as a 120 mg subcutaneous injection every 4 weeks according to institutional protocol, with routine calcium (500–1000 mg/day) and vitamin D supplementation (≥400 IU/day). Intravenous bisphosphonates—zoledronic acid (4 mg), ibandronate (6 mg), or incadronate disodium (10 mg)—were used as standard regimens and were given every 3–4 weeks, with infusion parameters and dose adjustments determined by renal function and local practice. Switching from a bisphosphonate to denosumab was at the treating oncologist’s discretion, most commonly because of renal dysfunction, the occurrence of an on-treatment SRE, inadequate response, or patient preference. In the sequential group, the minimum documented duration of prior bisphosphonate treatment before switching was 17 days, and the minimum number of bisphosphonate administrations was 1. Timing of switching relative to the first SRE was evaluable in 22 patients: 2 switched to denosumab before their first SRE, whereas 20 switched after SRE occurrence. In the remaining patients, the timing of switching relative to first SRE could not be classified with sufficient certainty from the retrospective clinical records. Patients underwent regular clinical and radiological follow-up according to standard practice, with CT or MRI performed every 3–6 months or as clinically indicated. SREs were retrospectively identified from the electronic medical records, including clinical notes, radiological reports, and treatment records. Pathologic fractures and spinal cord compression were confirmed using radiological findings together with the corresponding clinical documentation, whereas radiotherapy to bone and surgery to bone were identified from treatment records. No formal independent adjudication committee was used.

Laboratory monitoring included serum calcium, renal function tests, and tumor markers. Adverse events were abstracted from medical records and graded according to the Common Terminology Criteria for Adverse Events (CTCAE), version 5.0. The primary endpoint was time to first on-treatment SRE, with the 12-month first on-treatment SRE rate reported as a clinically relevant summary measure. The secondary endpoint was safety, assessed as the incidence of prespecified adverse events. Kaplan–Meier and log-rank tests were used to compare SRE-free survival between groups, and Cox regression was applied to explore prognostic factors.

### 2.3. Statistical Analysis

Continuous variables were summarized as means with standard deviations (SDs) or medians with interquartile ranges (IQRs) and compared between groups using Student’s *t* test or the Mann–Whitney *U* test, as appropriate. Categorical variables were presented as counts and percentages and compared using the χ^2^ test or Fisher’s exact test. The primary analysis compared time to first on-treatment SRE between the initial denosumab and sequential bisphosphonate-to-denosumab groups using Kaplan–Meier methods, with differences assessed by the log-rank test. Patients without an event were censored at the date of last follow-up.

Univariable Cox proportional hazards regression was used to identify clinical factors associated with time to first SRE. Hazard ratios (HRs) with 95% confidence intervals (CIs) were calculated, and the proportional hazards assumption was evaluated using Schoenfeld residuals. Safety endpoints were descriptively compared between groups using χ^2^ or Fisher’s exact tests. Missing data was handled using complete-case analysis. A two-sided *p* < 0.05 was considered statistically significant.

All analyses were performed using SPSS Statistics version 27.0 (IBM Corp., Armonk, NY, USA) and R version 4.3.1 (R Foundation for Statistical Computing, Vienna, Austria).

## 3. Results

### 3.1. Patient Cohort and Baseline Characteristics

Between 1 January 2019 and 30 April 2024, a total of 165 patients were eligible for analysis, including 98 who received sequential bisphosphonate-to-denosumab therapy and 67 who received initial denosumab. Baseline characteristics are summarized in Table 1. Median age at bone-metastasis diagnosis was 54.7 years (IQR 45.7–62.1), and there was no significant difference between groups. The interval from initial breast cancer diagnosis to bone metastasis varied by stage: 30.9 months for stage I (n = 5), 50.2 months for stage II (n = 14), 44.8 months for stage III (n = 11), and 29.8 months for stage IV disease (n = 125), indicating that most bone metastases occurred within 3–5 years of diagnosis irrespective of stage.

The interval from bone-metastasis confirmation to BTA initiation was similar (median 0.9 months in both groups). Median follow-up, however, was significantly longer in the sequential group (22.5 vs. 11.3 months; *p* < 0.001), consistent with greater total BTA exposure (median duration 20.8 vs. 7.7 months) and a higher number of bisphosphonate doses before switching to denosumab (median 10 doses). The number of denosumab administrations was similar in both groups (median 8 vs. 9). Distributions of surgery for the primary tumor, histological type, disease stage, and visceral metastases were balanced, while molecular subtype showed non-significant differences (HR+/HER2− 76.3% vs. 66.7%; triple-negative 3.2% vs. 12.1% in sequential vs. initial denosumab; *p* = 0.067). At the time of bone-metastasis diagnosis, 46 of 165 patients (27.9%) had at least one synchronous SRE, with a higher proportion in the initial denosumab group than in the sequential group (25/67 [37.3%] vs. 21/98 [21.4%]; *p* = 0.040). Among these synchronous events, bone radiotherapy (39.1%), pathological fractures (32.6%), and bone surgery (17.4%) were most frequent, and no cases of spinal cord compression were documented. Additional baseline characteristics, including nodal status and site of first recurrence, are provided in Table S1.

### 3.2. SREs After Bone-Metastasis Diagnosis

**Figure 2:**
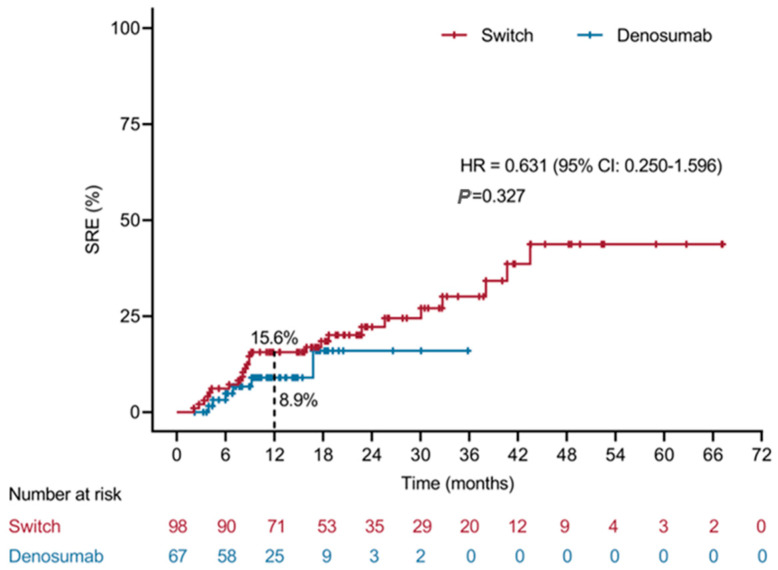
Kaplan–Meier curves for time to first SRE after bone-metastasis diagnosis in patients receiving sequential bisphosphonate-to-denosumab therapy versus initial denosumab. “Switch” denotes the sequential bisphosphonate-to-denosumab group, and “Denosumab” denotes the initial denosumab group. The 12-month cumulative SRE rate was 15.6% in the sequential group and 8.9% in the initial denosumab group (HR = 0.631, 95% CI 0.250–1.596; *p* = 0.327).

During follow-up after bone-metastasis diagnosis, 31 of 165 patients (18.8%) experienced at least one SRE. The incidence was significantly higher in the sequential bisphosphonate-to-denosumab group (25/98, 25.5%) than in the initial denosumab group (6/67, 9.0%; *p* = 0.008) (Table 2). First SREs were mainly bone radiotherapy (15/31, 48.4%) and pathological fracture (13/31, 41.9%), while bone surgery occurred in three patients (9.7%); no spinal-cord compression was observed. Repeat SREs were uncommon (4/31, 12.9%) and occurred exclusively in the sequential group. Kaplan–Meier analysis showed no statistically significant difference in time to first SRE between treatment groups (HR = 0.631, 95% CI 0.250–1.596; *p* = 0.327) (Figure 2). Additionally, malignancy-associated hypercalcemia was most frequent with bisphosphonates (8.3 events per 100 patient-years, sequential group), dropping to 5.4 post-denosumab switch and 1.4 events per 100 patient-years in the initial denosumab group (Table S2).

### 3.3. On-Treatment SREs After BTA Initiation

After initiation of BTA therapy, 28 of 165 patients (17.0%) developed on-treatment SREs. The proportion of patients with ≥1 on-treatment SREs was higher in the sequential bisphosphonate-to-denosumab group than in the initial denosumab group (25/98 [25.5%] vs. 3/67 [4.7%]; *p* < 0.001; Table 3). Among the 28 patients who developed an on-treatment SRE, the first event was bone radiotherapy in 14 (50.0%), pathological fracture in 13 (46.4%), and bone surgery in one (3.6%); no cases of spinal cord compression were observed. Multiple SREs occurred in three patients, all within the sequential group (3/25 [12.0%] vs. 0/3 [0%]; *p* = 0.005).

**Figure 3:**
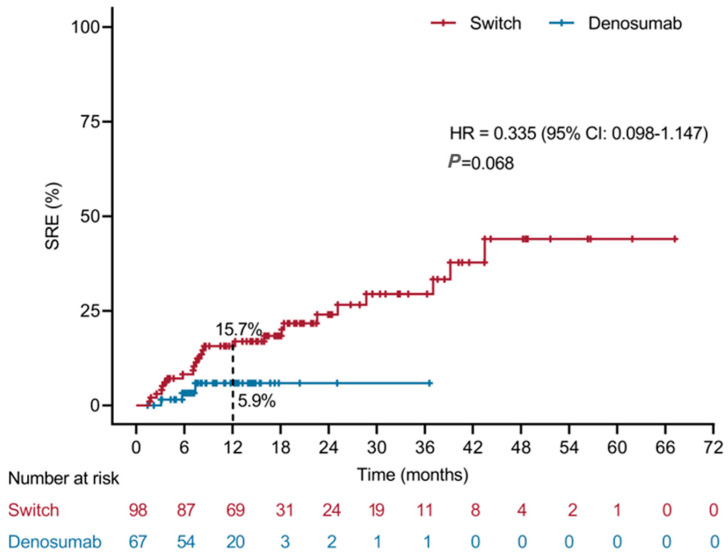
Kaplan–Meier curves for time to first on-treatment SRE after BTA initiation. “Switch” denotes the sequential bisphosphonate-to-denosumab group, and “Denosumab” denotes the initial denosumab group. The 12-month first on-treatment SRE rate was 15.7% for the sequential bisphosphonate-to-denosumab group and 5.9% for the initial denosumab group (HR = 0.335, 95% CI 0.098–1.147; *p* = 0.068). The reported *p*-value refers to the overall between-group comparison from the time-to-event analysis.

Kaplan–Meier analysis of time to first on-treatment SRE showed a numerically lower risk in the initial denosumab group, although the between-group difference did not reach statistical significance (HR = 0.335, 95% CI 0.098–1.147; *p* = 0.068) (Figure 3).

### 3.4. Exploratory Analysis of Factors Associated with SRE Risk

**Figure 4:**
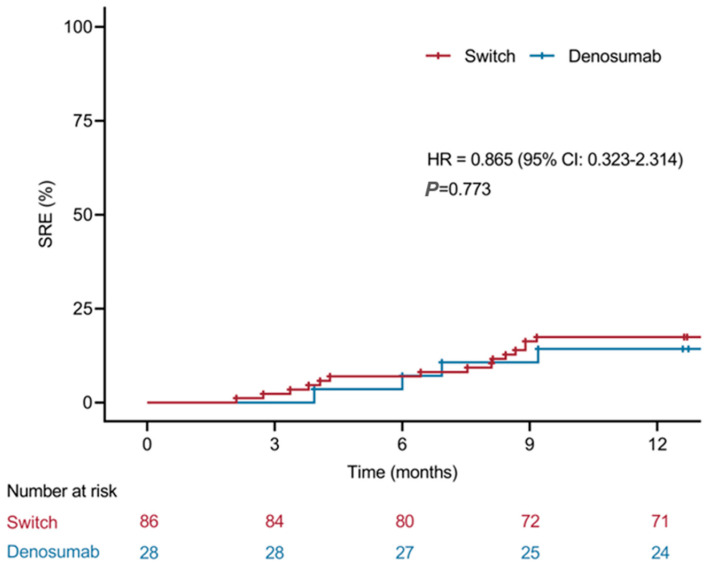
Kaplan–Meier curves for time to first on-treatment SRE according to treatment group. “Switch” denotes the sequential bisphosphonate-to-denosumab group, and “Denosumab” denotes the initial denosumab group. No significant difference was observed between sequential bisphosphonate-to-denosumab and initial denosumab groups (HR = 0.865, 95% CI 0.323––2.314; *p* = 0.773).

In an exploratory Kaplan–Meier analysis comparing the sequential bisphosphonate-to-denosumab group and the initial denosumab group, no significant difference in time to the first on-treatment SRE was observed (HR = 0.865, 95% CI 0.323–2.314; *p* = 0.773) (Figure 4). Given the relatively early initiation of BTA in both cohorts and the limited number of SREs during follow-up, this analysis was probably underpowered to detect modest differences in risk. In univariable Cox regression (Table 4), disease stage and line of systemic therapy were associated with SRE risk. Compared with stage I disease, stage IV disease was paradoxically associated with a lower hazard of SREs (HR 0.192, 95% CI 0.056–0.657; *p* = 0.009). Receipt of second-line systemic therapy significantly increased SRE risk (HR 2.651, 1.160–6.057; *p* = 0.021), whereas treatment with ≥3 lines was not significantly associated with risk. Other clinical covariates, including age, visceral metastasis, bone lesion burden, and treatment group, were not significantly associated with SRE risk in univariable analyses.

### 3.5. Safety

Because treatment duration differed across treatment periods and groups, adverse events were summarized as exposure-adjusted event rates per 100 patient-years. During the bisphosphonate treatment period in the sequential group, renal impairment, hypocalcemia, and ONJ occurred at rates of 6.6, 7.5, and 1.7 events per 100 patient-years, respectively. During the denosumab treatment period in the sequential group, the corresponding rates were 8.2, 5.4, and 0.9 events per 100 patient-years, whereas in the initial denosumab group they were 8.4, 11.2, and 0 events per 100 patient-years, respectively. Most adverse events were grade 1 and clinically manageable, with few grade 2 events observed. These exposure-adjusted safety data should be descriptively interpreted and do not establish a clear safety advantage of one strategy over another (Table 5).

## 4. Discussion

Randomized trials established the superiority of denosumab over zoledronic acid for delaying first SREs in metastatic breast cancer and other solid tumors, but these studies evaluated patients at the time of BTA initiation and did not explore sequential use [16,17,18]. Subsequent registry and claims-based analyses have yielded divergent results: European and North American cohorts have reported either comparable outcomes between sequential and initial strategies [10,19] or a higher SRE risk when denosumab is introduced after an initial period of bisphosphonate therapy [20,21,22]. Against this background, our single-center retrospective cohort helps to clarify the potential impact of treatment timing. The 12-month incidence of first on-treatment SRE in the denosumab-first group was broadly consistent with the efficacy signal reported in pivotal randomized trials, although cross-study comparisons should be interpreted with caution [16,17]. This interpretation is further supported by more recent evidence syntheses and real-world studies [23,24,25,26].

Moreover, within our cohort, initiating denosumab as the first bone-modifying agent was associated with a markedly lower incidence of first on-treatment SREs compared with a sequential bisphosphonate-to-denosumab strategy (4.7% vs. 25.5%), and the Kaplan–Meier curves suggested a numerically lower 12-month SRE risk (5.9% vs. 15.7%; HR 0.34), despite shorter follow-up in the initial group. Although these differences did not reach conventional statistical significance because of limited power, the consistency in effect direction across multiple analyses indicates that the timing of denosumab initiation is likely an important determinant of skeletal outcomes in routine practice. These findings complement guideline recommendations that advise starting a bone-modifying agent as soon as bone metastases are diagnosed, irrespective of symptoms [27]. Randomized phase 3 trials established denosumab as at least non-inferior and, in breast cancer, superior to zoledronic acid in delaying first and subsequent SREs, with comparable survival and a more favorable renal safety profile [16,28]. Subsequent systematic reviews and meta-analyses have confirmed that denosumab reduces SRE risk by roughly 20–25% compared with intravenous bisphosphonates across solid tumors, including breast cancer [23,29]. However, almost all pivotal trials randomized patients at BTA initiation and did not address the increasingly common real-world strategy of starting with a bisphosphonate and switching later to denosumab. Registry and claims-based analyses from Europe and North America have suggested heterogeneous patterns of BTA use and switching, with some cohorts reporting similar outcomes between agents and others indicating higher SRE rates when denosumab is introduced late in the disease course [22,30,31]. Emerging real-world data presented in abstract form likewise indicate that first-line denosumab may provide superior SRE prevention compared with sequential bisphosphonate-to-denosumab therapy in metastatic breast cancer [32]. Our study adds to this evidence base in an Asian population, using detailed chart-confirmed SRE adjudication. The differential effect between strategies is biologically plausible. Nitrogen-containing bisphosphonates require incorporation into actively remodeling bone and induce osteoclast apoptosis via disruption of the mevalonate pathway, a process that may be less efficient in rapidly progressive osteolytic lesions where osteoclast turnover is very high [27]. Denosumab, by contrast, neutralizes circulating and membrane-bound RANKL, leading to near-complete and rapid suppression of bone-resorption markers within days of administration [33]. If denosumab is introduced only after several months of bisphosphonate therapy, micro-fracturing, cortical thinning, and structural destabilization may already have occurred and may be only partly reversible. The early separation of Kaplan–Meier curves in favor of the denosumab-first group in our cohort is consistent with this kinetic hypothesis and echoes epidemiological data showing that SRE risk is highest in the first year after bone-metastasis diagnosis [27].

In our cohort, the median interval from radiological confirmation of bone metastases to BTA initiation was only 0.9 months, and most patients started treatment within three months. This timing is consistent with international guidelines recommending early use of bone-modifying agents once bone metastases are diagnosed [4]. Real-world studies have also used the first three months as a practical cut-off for “early” versus “delayed” BTA initiation [10,11]. Our exploratory Cox analyses provide additional insight into disease dynamics. Second-line systemic therapy was associated with a significantly higher hazard of SREs, suggesting that skeletal complications cluster in patients with treatment-refractory disease and more aggressive tumor biology. Conversely, stage IV disease at initial breast-cancer diagnosis was paradoxically associated with a lower SRE hazard, which is likely driven by competing mortality: patients with rapidly fatal disease may die before living long enough to experience on-treatment SREs. Similar patterns have been reported in large population-based cohorts, in which death before SREs is a major competing event that complicates interpretation of crude SRE rates. These findings underscore that SREs are not merely a function of tumor stage but arise from the interplay between tumor burden, systemic treatment history, and survival. Future studies using competing-risk models and target-trial emulation could more accurately estimate the causal effect of BTA strategy on skeletal outcomes [34].

The safety profile observed in this cohort aligns with the known pharmacology of BTAs. After adjustment for treatment exposure, renal impairment rates were numerically similar across treatment groups, while hypocalcemia showed some numerical variation and was highest in the initial denosumab group. These descriptive findings should be cautiously interpreted and do not establish a clear comparative safety advantage of one strategy over another. Nevertheless, the overall pattern remains broadly consistent with the established class-specific safety profiles reported in previous trials and meta-analyses, in which intravenous bisphosphonates were associated with greater renal toxicity and denosumab with a higher risk of hypocalcemia [16,28,35]. In our cohort, medication-related osteonecrosis of the jaw (MRONJ) was uncommon, occurring in three patients (3.0%) during prolonged bisphosphonate treatment in the sequential group and in no patients receiving upfront denosumab. The low absolute incidence probably reflects limited cumulative exposure, systematic dental screening, and the relatively short follow-up. Nonetheless, longer-term series indicate that MRONJ risk increases with the duration of antiresorptive therapy, particularly with sequential or high cumulative dosing, and this must be weighed against the benefit of prolonged SRE prevention [33,36].

Our findings also have health–economic implications. With recent adjustments in pricing and reimbursement policies, including the incorporation of denosumab into national insurance schemes in China, the budget impact of choosing denosumab over bisphosphonates is changing. A recent pharmacoeconomic analysis suggested that denosumab can be cost-effective compared with zoledronic acid in certain settings, mainly through reductions in SRE-related hospitalizations and preserved renal function [37]. Our data showing fewer SREs and no excess toxicity with initial denosumab supports the biological premise of these models.

Several limitations warrant caution. First, this was a retrospective, non-randomized study; and both the choice of BTA and timing of switch were determined by treating physicians in routine clinical practice. As a result, the sequential group may have been enriched for patients with greater clinical complexity during prior bisphosphonate treatment, including renal dysfunction, on-treatment SREs, or other reasons prompting treatment modification. This introduces potential confounding by indication that may bias against the sequential strategy. Second, follow-up duration was longer in the sequential group, increasing the opportunity to observe SREs despite our use of time-to-event methods. Third, the number of SREs was modest, limiting statistical power and restricting our Cox modeling to univariable analyses; residual confounding and type-II error are therefore likely. In particular, given the modest number of on-treatment SREs, especially in the initial denosumab group, this study may have had limited power to detect modest between-group differences and to support robust multivariable adjustment for several clinically relevant covariates. Fourth, competing-risk methods were not applied, so the influence of early death on SRE incidence could not be fully quantified. Finally, we lacked systematic data on pain scores, functional status, and more specific markers of bone formation, bone resorption, or inflammatory activity, such as P1NP (type I procollagen amino-terminal peptide), osteocalcin and CTX (type I collagen carboxy-terminal peptide)/NTX (type I collagen amino-terminal peptide), which may provide additional clinically relevant information for a more holistic assessment of disease burden and treatment benefit beyond radiologically defined SREs.

Despite these caveats, the overall pattern of evidence supports a clinically meaningful advantage of initiating denosumab at the time of bone-metastasis diagnosis rather than after a period of bisphosphonate exposure. In line with prior work, our data reinforces the central role of preventing the first SRE, given that subsequent events often cluster, drive hospital admissions, and are associated with deterioration in mobility and quality of life [27]. From a clinical perspective, denosumab should be strongly considered as the preferred first-line bone-modifying agent for patients with newly diagnosed breast-cancer bone metastases, particularly in those with renal impairment, extensive osteolytic disease, or high early SRE risk. Future prospective, multicenter studies using causal-inference approaches, predefined SRE adjudication, and incorporation of patient-reported outcomes will be essential to confirm whether early denosumab initiation can consistently translate into fewer skeletal complications, improved quality of life, and acceptable long-term safety across diverse healthcare settings.

## 5. Conclusions

In this retrospective single-center cohort of patients with breast cancer and bone metastases, use of denosumab as the initial bone-targeting agent was associated with a substantially lower risk and later occurrence of first on-treatment skeletal-related events than a sequential bisphosphonate-to-denosumab strategy, despite shorter follow-up in the initial group. Safety outcomes were consistent with the expected profiles of both drug classes, with no new safety signals observed. Taken together, these findings support initial denosumab as a preferable bone-modifying strategy in routine practice, particularly for patients at high risk of early skeletal complications. Prospective multicenter studies using robust causal-inference methods and incorporating patient-reported outcomes are needed to confirm these observations and define the long-term clinical and economic impact of early denosumab initiation.

## Figures and Tables

**Table 1:** Clinical and pathological characteristics of 165 patients with BC.

Characteristic	Overall (n = 165)	Sequential Bisphosphonate-to-Denosumab (n = 98)	Initial Denosumab (n = 67)	*p* Value
**Age at breast cancer diagnosis, years**				
Mean ± SD	49.8 ± 11.3	50.1 ± 10.6	49.4 ± 12.3	
Median (IQR)	49.0 (42.0, 58.0)	50.0 (42.0, 58.0)	48.0 (39.0, 58.0)	0.588
Min, max	25.0, 79.0	27.0, 79.0	25.0, 77.0	
**Age at bone-metastasis diagnosis, years**				
Mean ± SD	53.9 ± 12.0	54.3 ± 11.8	53.4 ± 12.5	
Median (IQR)	54.7 (45.7, 62.1)	55.0 (46.0, 62.3)	54.6 (42.0, 60.8)	0.531
Min, max	27.1, 91.7	28.0, 91.7	27.1, 81.5	
**Histological type, n (%)**				>0.999
Invasive carcinoma	151 (95.0)	88 (94.6)	63 (95.5)	
Invasive ductal carcinoma	71 (44.7)	38 (40.9)	33 (50.0)	
Invasive lobular carcinoma	12 (7.5)	9 (9.7)	3 (4.5)	
Other	68 (42.8)	41 (44.1)	27 (40.9)	
Non-invasive carcinoma	8 (5.0)	5 (5.4)	3 (4.5)	
N, missing	165, 6	98, 5	67, 1	
**Stage, n (%)**				0.453
I	5 (3.2)	2 (2.2)	3 (4.7)	
II	14 (9.0)	10 (11.0)	4 (6.3)	
III	11 (7.1)	8 (8.8)	3 (4.7)	
IV	125 (80.6)	71 (78.0)	54 (84.4)	
N, missing	165, 10	98, 7	67, 3	
**Histological grade, n (%)**				0.475
I	2 (1.9)	2 (3.4)	0	
II	52 (50.0)	31 (52.5)	21 (46.7)	
III	50 (48.1)	26 (44.1)	24 (53.3)	
N, missing	165, 61	98, 39	67, 24	
**Molecular subtype, n (%)**				0.067
HR+HER2−	115 (72.3)	71 (76.3)	44 (66.7)	
HR+HER2+	15 (9.4)	7 (7.5)	8 (12.1)	
HR−HER2+	11 (6.9)	6 (6.5)	5 (7.6)	
TNBC	11 (6.9)	3 (3.2)	8 (12.1)	
HR−	2 (1.3)	1 (1.1)	1 (1.5)	
HR+	5 (3.1)	5 (5.4)	0	
N, missing	165, 6	98, 5	67, 1	
**Bone metastasis at initial diagnosis, n (%)**				0.359
Yes	56 (33.9)	36 (36.7)	20 (29.9)	
No	109 (66.1)	62 (63.3)	47 (70.1)	
**Line of therapy, n (%)**				<0.001
1st-line	103 (62.4)	50 (51.0)	53 (79.1)	
2nd-line	32 (19.4)	22 (22.4)	10 (14.9)	
≥3rd-line	30 (18.2)	26 (26.5)	4 (6.0)	
**Number of bone metastases, n (%)**				
<3	72 (43.6)	37 (37.8)	35 (52.2)	0.079
≥3	93 (56.4)	61 (62.2)	32 (47.8)	
<5	106 (64.2)	62 (63.3)	44 (65.7)	0.869
≥5	59 (35.8)	36 (36.7)	23 (34.3)	
**Bone metastasis site, n (%)**				0.168
Non-weight-bearing bone	31 (19.0)	15 (15.5)	16 (24.2)	
Weight-bearing bone	34 (20.9)	18 (18.6)	16 (24.2)	
Mixed	98 (60.1)	64 (66.0)	34 (51.5)	
N, missing	165, 2	98, 1	67, 1	
**Follow-up time, months**				
Mean ± SD	21.9 ± 15.2	28.1 ± 15.9	12.8 ± 7.6	
Median (IQR)	17.3 (11.1, 29.4)	22.5 (16.5, 37.7)	11.3 (7.9, 15.8)	<0.001
Min, Max	2.2, 67.2	4.1, 67.2	2.2, 40.6	
<12 months	52 (31.5)	13 (13.3)	39 (58.2)	<0.001
≥12 months	113 (68.5)	85 (86.7)	28 (41.8)	
**Time from bone-metastasis diagnosis to BTA initiation, months**				
Mean ± SD	2.1 ± 3.8	1.7 ± 3.0	2.7 ± 4.7	
Median (IQR)	0.9 (0.3, 1.8)	0.9 (0.3, 1.7)	0.9 (0.3, 2.3)	0.513
Min, Max	0, 23.3	0.03, 18.6	0.03, 23.3	
**BTA treatment duration (from initiation to end), months**				
Mean ± SD	19.4 ± 14.9	26.0 ± 15.7	9.8 ± 5.8	
Median (IQR)	15.2 (8.0, 24.2)	20.8 (14.5, 36.3)	7.7 (6.5, 12.8)	<0.001
Min, Max	1.4, 67.2	2.3, 67.2	1.4, 36.6	
**Time from breast cancer diagnosis to bone metastasis, Median (range), months**				
Stage I	30.9 (0.3, 56.4)	28.4 (0.3, 56.4)	30.9 (4.3, 40.5)	
Stage II	50.2 (1.4, 178.6)	35.8 (1.4, 178.6)	79.4 (17.7, 140.8)	
Stage III	44.8 (14.2, 197.9)	39.4 (17.0, 197.9)	44.8 (14.2, 64.4)	
Stage IV	29.8 (0, 217.3)	17.8 (0, 217.3)	40.5 (0, 199.4)	
**Duration of bisphosphonate treatment, months**				
Mean ± SD	14.1 ± 14.0	14.1 ± 14.0	-	
Median (IQR)	9.7 (2.8, 22.2)	9.7 (2.8, 22.2)	-	
Min, Max	0.03, 62.0	0.03, 62.0	-	
**Duration of denosumab treatment, months**				
Mean ± SD	9.0 ± 5.8	8.4 ± 5.8	9.9 ± 5.7	
Median (IQR)	7.3 (6.1, 11.4)	7.0 (5.8, 9.7)	8.0 (6.6, 12.6)	
Min, Max	0.03, 36.6	0.03, 33.9	1.4, 36.6	
**Number of bisphosphonate administrations**				
Mean ± SD	12.0 ± 9.6	12.0 ± 9.6	-	
Median (IQR)	10.0 (4.0, 19.0)	10.0 (4.0, 19.0)	-	
Min, Max	1, 41	1, 41	-	
**Number of denosumab administrations**				
Mean ± SD	9.4 ± 4.7	8.6 ± 3.9	10.5 ± 5.5	
Median (IQR)	8.0 (7.0, 11.0)	8.0 (7.0, 10.0)	9.0 (7.5, 13.0)	
Min, Max	1, 36	1, 22	1, 36	
**SREs at bone metastasis diagnosis**				
**incidence of SREs, n (%)**				0.040
Yes	46 (27.9)	21 (21.4)	25 (37.3)	
No	119 (72.1)	77 (78.6)	42 (62.7)	
**Type of SRE, n (%)**	n = 46	n = 21	n = 25	
Pathological fracture	15 (32.6)	9 (42.9)	6 (24.0)	
Bone radiotherapy	18 (39.1)	7 (33.3)	11 (44.0)	
Bone surgery	8 (17.4)	3 (14.3)	5 (20.0)	
Spinal cord compression	0	0	0	
Fracture + surgery	5 (10.9)	2 (9.5)	3 (12.0)	

**Table 2:** Incidence and types of SREs after bone metastasis diagnosis.

SREs After Bone Metastasis	Overall (n = 165)	Sequential Bisphosphonate-to-Denosumab (n = 98)	Initial Denosumab (n = 67)	*p* Value
**SRE occurrence within 12-month follow-up, n (%)**	n = 114	n = 86	n = 28	>0.999
Yes	19 (16.7)	15 (17.4)	4 (14.3)	
No	95 (83.3)	71 (82.6)	24 (85.7)	
**Incidence of SREs, n (%)**				**0.008**
Yes	31 (18.8)	25 (25.5)	6 (9.0)	
No	134 (81.2)	73 (74.5)	61 (91.0)	
**Type of first SRE, n (%)**	n = 31	n = 25	n = 6	
Pathological fracture	13 (41.9)	10 (40.0)	3 (50.0)	
Bone radiotherapy	15 (48.4)	14 (56.0)	1 (16.7)	
Bone surgery	3 (9.7)	1 (4.0)	2 (33.3)	
Spinal cord compression	0	0	0	
**Cumulative number of SREs, n (%)**	n = 31	n = 25	n = 6	**0.005**
1 event	27 (87.1)	21 (84.0)	6 (100.0)	
≥2 events	4 (12.9)	4 (16.0)	0	
Median time from bone-metastasis diagnosis to first SRE, 95%CI	NR (40.7–NR)	NR (40.7–NR)	NR (NR–NR)	
**12-month cumulative SRE rate, % (95% CI)**	13.3 (7.7–18.6)	15.6 (8.0–22.6)	8.9 (1.0–16.3)	

Abbreviations: NR, not reached.

**Table 3:** Incidence and types of SREs after BTA initiation.

SREs After BTA Initiation	Overall (n = 165)	Sequential Bisphosphonate-to-Denosumab (n = 98)	Initial Denosumab (n = 67)	*p* Value
**SRE occurrence within 12 months after BTA initiation, n (%)**	n = 101	n = 81	n = 20	0.513
Yes	17 (16.8)	15 (18.5)	2 (10.0)	
No	84 (83.2)	66 (81.5)	18 (90.0)	
**Incidence of SREs, n (%)**				**<0.001**
Yes	28 (17.0)	25 (25.5)	3 (4.7)	
No	137 (83.0)	73 (74.5)	64 (95.3)	
**Type of first SRE, n (%)**	n = 28	n = 25	n = 3	
Pathological fracture	13 (46.4)	10 (40.0)	3 (100.0)	
Bone radiotherapy	14 (50.0)	14 (56.0)	0	
Bone surgery	1 (3.6)	1 (4.0)	0	
Spinal cord compression	0	0	0	
**Cumulative number of SREs, n (%)**	n = 28	n = 25	n = 3	**0.005**
1 event	25 (89.3)	22 (88.0)	3 (100.0)	
≥2 events	3 (10.7)	3 (12.0)	0	
Median time from BTA initiation to first SRE, 95% CI	NR (43.5–NR)	NR (39.2–NR)	NR (NR–NR)	
**12-month first on-treatment SRE rate, %** (95% CI)	12.5 (6.8–17.7)	15.7 (8.1–22.7)	5.9 (0–12.3)	

Abbreviations: NR, not reached.

**Table 4:** Univariate analysis of risk factors for SREs.

Characteristic	No SRE (n = 134)	SRE (n = 31)	HR (95% CI)	*p* Value
**Age at bone-metastasis diagnosis, years**				
Mean ± SD	53.7 ± 12.6	55.0 ± 9.4		
Median (IQR)	54.8 (43.7, 62.1)	54.6 (46.8, 62.3)	1.007 (0.979–1.036)	0.620
Min, max	27.1, 91.7	34.0, 70.0		
**Surgery for primary tumor, n (%)**				
Yes	86 (64.2)	25 (80.6)	Ref.	-
No	48 (35.8)	6 (19.4)	0.412 (0.169–1.006)	0.052
**Histological type, n (%)**				
Invasive carcinoma	123 (94.6)	28 (96.6)	Ref.	-
Non-invasive carcinoma	7 (5.4)	1 (3.4)	0.677 (0.092–4.991)	0.702
N, missing	134, 4	31, 2		
**Stage, n (%)**				
I	2 (1.6)	3 (10.0)	Ref.	-
II	12 (9.6)	2 (6.7)	0.227 (0.038–1.361)	0.105
III	7 (5.6)	4 (13.3)	0.542 (0.121–2.435)	0.424
IV	104 (83.2)	21 (70.0)	0.192 (0.056–0.657)	**0.009**
N, missing	134, 9	31, 1		
**Histological grade, n (%)**				
I	1 (1.1)	1 (5.9)	Ref.	-
II	43 (49.4)	9 (52.9)	0.324 (0.040–2.645)	0.293
III	43 (49.4)	7 (41.2)	0.324 (0.040–2.645)	0.254
N, missing	134, 47	31, 14		
**Lymph node metastasis at initial diagnosis, n (%)**				
Yes	96 (71.6)	21 (67.7)	Ref.	-
No	38 (28.4)	10 (32.3)	1.122 (0.527–2.388)	0.765
**Bone metastasis at initial diagnosis, n (%)**				
Yes	46 (34.3)	10 (32.3)	Ref.	-
No	88 (65.7)	21 (67.7)	1.175 (0.552–2.502)	0.675
**First recurrence/metastasis site, n (%)**				
Bone	117 (88.0)	29 (93.5)	Ref.	-
Non-bone	16 (12.0)	2 (6.5)	0.588 (0.140–2.469)	0.468
N, missing	134, 1	31, 0		
**Line of therapy, n (%)**				
1st-line	91 (67.9)	12 (38.7)	Ref.	-
2nd-line	21 (15.7)	11 (35.5)	2.651 (1.160–6.057)	**0.021**
≥3rd-line	22 (16.4)	8 (25.8)	1.539 (0.621–3.814)	0.352
**Visceral metastasis, n (%)**				
Yes	71 (53.0)	14 (45.2)	Ref.	-
No	63 (47.0)	17 (54.8)	1.319 (0.650–2.679)	0.444
**Number of bone metastases, n (%)**				
<3	56 (41.8)	16 (51.6)	Ref.	-
≥3	78 (58.2)	15 (48.4)	0.595 (0.293–1.207)	0.150
<5	84 (62.7)	22 (71.0)	Ref.	-
≥5	50 (37.3)	9 (29.0)	0.748 (0.344–1.628)	0.465
**Bone metastasis site, n (%)**				
Weight-bearing	26 (19.5)	8 (26.7)	Ref.	-
Non-weight-bearing	28 (21.1)	3 (10.0)	0.376 (0.100–1.420)	0.149
Mixed	79 (59.4)	19 (63.3)	0.735 (0.321–1.682)	0.466
N, missing	134, 1	31, 1		
**Treatment regimen, n (%)**				
Sequential bisphosphonate-to-denosumab	73 (54.5)	25 (80.6)	Ref.	-
Denosumab monotherapy	61 (45.5)	6 (19.4)	0.631 (0.250–1.596)	0.331

**Table 5:** Exposure-adjusted adverse-event rates (events per 100 patient-years).

Adverse Event	Sequential Bisphosphonate-to-Denosumab						Initial Denosumab n = 67		
	Bisphosphonate Period n = 98			Denosumab Period n = 98					
	Any Grade	Grade 1	Grade 2	Any Grade	Grade 1	Grade 2	Any Grade	Grade 1	Grade 2
Renal impairment	8 (6.6)	8 (6.6)	0	9 (8.2)	9 (8.2)	0	6 (8.4)	6 (8.4)	0
Hypocalcemia	9 (7.5)	7 (5.8)	2 (1.7)	6 (5.4)	5 (4.5)	1 (0.9)	8 (11.2)	6 (8.4)	2 (2.8)
ONJ	2 (1.7)	–	–	1 (0.9)	–	–	0	0	0

Footnote: Values are presented as number of events (events per 100 patient-years). Event rates were calculated as the number of adverse events divided by the total follow-up time in patient-years and multiplied by 100. In the sequential group, adverse events were attributed to the treatment period during which they occurred.

## Data Availability

All data generated or analyzed during this study are included in this published article.

## Supplementary Materials

The following supporting information can be downloaded at: https://www.mdpi.com/article/10.3390/cancers18081222/s1, Table S1: Clinical and pathological characteristics of 165 patients with BC; Table S2: Exposure-adjusted incidence of malignancy-associated hypercalcemia by treatment group.

## Abbreviations

BTA	bone-targeting agent
CI	confidence interval
CTCAE	Common Terminology Criteria for Adverse Events
HER2	human epidermal growth factor receptor 2
HR	hazard ratio
IQR	interquartile range
MRONJ	medication-related osteonecrosis of the jaw
RANKL	receptor activator of nuclear factor-κB ligand
SRE	skeletal-related event
