# Supplementary material for: Initial Denosumab Versus Sequential Bisphosphonate-to-Denosumab for Prevention of Skeletal-Related Events in Breast Cancer with Bone Metastases: A Retrospective, Single-Center Study
Source: Cancers (Basel). 2026 Apr 12;18(8):1222. doi: 10.3390/cancers18081222 (PMC13115166; doi:10.3390/cancers18081222)
Supplement: Supplementary file 1 [file cancers-18-01222-s001.zip › cancers-4196365-supplementary.pdf]

Table S1. Clinical and pathological characteristics of 165 patients with BC.

Characteristic	Overall (n=165)	Sequential Bisphosphonate-to-Denosumab (n=98)	Initial-denosumab (n=67)	*P value*
**Age at breast cancer diagnosis, years**				
Mean ± SD	49.8±11.3	50.1±10.6	49.4±12.3	
Median (IQR)	49.0(42.0,58.0)	50.0(42.0,58.0)	48.0(39.0,58.0)	0.588
Min, max	25.0,79.0	27.0,79.0	25.0,77.0	
≤60	136(82.4)	80(81.6)	56(83.6)	0.747
>60	29(17.6)	18(18.4)	11(16.4)	
≤55	112(67.9)	66(67.3)	46(68.7)	0.860
>55	53(32.1)	32(32.7)	21(31.3)	
≤50	87(52.7)	50(51.0)	37(55.2)	0.595
>50	78(47.3)	48(49.0)	30(44.8)	
**Age at bone metastasis diagnosis, years**				
Mean ± SD	53.9±12.0	54.3±11.8	53.4±12.5	
Median (IQR)	54.7(45.7,62.1)	55.0(46.0,62.3)	54.6(42.0,60.8)	0.531
Min, max	27.1,91.7	28.0,91.7	27.1,81.5	
≤60	116(70.3)	66(67.3)	50(74.6)	0.315
>60	49(29.7)	32(32.7)	17(25.4)	
≤55	91(55.2)	51(52.0)	40(59.7)	0.331
>55	74(44.8)	47(48.0)	27(40.3)	
≤50	66(40.0)	39(39.8)	27(40.3)	0.948
>50	99(60.0)	59(60.2)	40(59.7)	
**Surgery for primary tumor, n (%)**				0.754
Yes	111(67.3)	65(66.3)	46(68.7)	
No	54(32.7)	33(33.7)	21(31.3)	
**Lymph node metastasis at initial diagnosis, n (%)**				0.864
Yes	117(70.9)	69(70.4)	48(71.6)	
No	48(29.1)	29(29.6)	19(28.4)	
**Visceral metastasis, n (%)**				0.870
Yes	85(51.5)	51(52.0)	34(50.7)	
Lung	54(32.7)	32(32.7)	22(32.8)	
Liver	38(23.0)	20(20.4)	18(26.9)	
Stomach	1(0.6)	1(1.0)	0	
No	80(48.5)	47(48.0)	33(49.3)	
**First recurrence/metastatic site, n (%)**				
Bone	146(88.5)	84(86.6)	62(92.55)	
Lymph nodes	22(13.3)	14(14.4)	8(11.9)	
Liver	21(12.7)	9(9.3)	12(17.9)	
Lung	26(15.8)	16(16.5)	10(14.9)	
Others	6(3.6)	4(4.1)	2(3.0)	
N, missing	165,1	98,1	67,0	

Table S2. Exposure-adjusted incidence of malignancy-associated hypercalcemia by treatment group.

Event	Sequential Bisphosphonate-to-Denosumab						Initial-denosumab n=67		
	Bisphosphonate period n=98			Denosumab period n=98					
	Any grade	Grade 1	Grade 2	Any grade	Grade 1	Grade 2	Any grade	Grade 1	Grade 2
Malignancy-associated hypercalcemia	10(8.3)	--	--	6(5.4)	--	--	1(1.4)	--	--
